# Sustained Improvement in Health‐Related Quality of Life in Transplant‐Ineligible Newly Diagnosed Multiple Myeloma Treated With Daratumumab, Lenalidomide, and Dexamethasone: MAIA Final Analysis of Patient‐Reported Outcomes

**DOI:** 10.1111/ejh.14392

**Published:** 2025-02-14

**Authors:** Aurore Perrot, Thierry Facon, Torben Plesner, Saad Z. Usmani, Shaji Kumar, Nizar J. Bahlis, Cyrille Hulin, Robert Z. Orlowski, Hareth Nahi, Peter Mollee, Karthik Ramasamy, Murielle Roussel, Arnaud Jaccard, Michel Delforge, Lionel Karlin, Bertrand Arnulf, Ajai Chari, George Wang, Niodita Gupta‐Werner, Shuchita Kaila, Huiling Pei, Kathryn Matt, Katharine S. Gries, Robin Carson, Fredrik Borgsten, Katja Weisel

**Affiliations:** ^1^ Centre Hospitalier Universitaire de Toulouse Service d'Hématologie Toulouse France; ^2^ University of Lille, CHU Lille Lille France; ^3^ Vejle Hospital and University of Southern Denmark Vejle Denmark; ^4^ Memorial Sloan Kettering Cancer Center New York New York USA; ^5^ Mayo Clinic Rochester Minnesota USA; ^6^ University of Calgary, Arnie Charbonneau Cancer Research Institute Calgary Alberta Canada; ^7^ Hôpital Haut Leveque University Hospital Pessac France; ^8^ The University of Texas MD Anderson Cancer Center Houston Texas USA; ^9^ Karolinska University Hospital at Huddinge Stockholm Sweden; ^10^ Princess Alexandra Hospital and University of Queensland Medical School Brisbane Queensland Australia; ^11^ Oxford University Hospitals, NHS Foundation Trust Oxford UK; ^12^ Centre Hospitalier Universitaire Limoges France; ^13^ University Hospital Leuven Leuven Belgium; ^14^ Centre Hospitalier Lyon Sud Pierre–Bénite France; ^15^ Immuno‐Hématologie Hospital Saint Louis, APHP Paris France; ^16^ Mount Sinai School of Medicine New York New York USA; ^17^ Johnson & Johnson Spring House Pennsylvania USA; ^18^ Johnson & Johnson Horsham Pennsylvania USA; ^19^ Johnson & Johnson Titusville New Jersey USA; ^20^ Johnson & Johnson Raritan New Jersey USA; ^21^ University Medical Center Hamburg‐Eppendorf Hamburg Germany

**Keywords:** elderly, frail, health‐related quality of life, multiple myeloma, patient‐reported outcomes

## Abstract

**Objectives:**

This final post hoc analysis evaluated patient‐reported outcomes from the Phase 3 MAIA study of daratumumab, lenalidomide, and dexamethasone (D‐Rd) versus lenalidomide and dexamethasone (Rd) after median 64.5‐month follow‐up in transplant‐ineligible patients with newly diagnosed multiple myeloma (NDMM), including patient subgroups.

**Methods:**

Key scales from the EORTC QLQ‐C30 (global health status [GHS], physical functioning, pain, and fatigue) were assessed. Scores were evaluated every 3 months for 1 year, then every 6 months until disease progression.

**Results:**

The intent‐to‐treat population (*n* = 737) included 46.3% frail, 35.4% 70 to < 75 years old, and 43.6% ≥ 75 years old. D‐Rd‐treated patients showed improvements from baseline that were sustained over 5 years in the intent‐to‐treat population and across subgroups by age, frailty, and bone lesions. Greater proportions of patients treated with D‐Rd versus Rd achieved minimally important changes for improvement at cycle 36 (year ~3) in GHS (odds ratio, 1.84 [95% CI, 1.16–2.91]), physical functioning (1.93 [1.18–3.14]), pain (1.41 [0.90–2.22]), and fatigue (2.00 [1.24–3.23]). Greater proportions of patients with bone lesions improved with D‐Rd versus Rd on GHS and physical functioning.

**Conclusions:**

In transplant‐ineligible patients with NDMM, D‐Rd improved health‐related quality of life over a 5‐year period versus Rd.

**Trial Registration:** ClinicalTrials.gov: NCT02252172

## 1 Introduction

Daratumumab was the first anti‐CD38 monoclonal antibody approved in newly diagnosed multiple myeloma (NDMM) and has demonstrated an overall survival benefit in three frontline regimens [1, 2, 3, 4, 5]. Although triplet and quadruplet regimens are associated with superior outcomes in the transplant‐ineligible population compared with doublet therapy [2, 6, 7], they are less likely to be used in elderly and/or frail patients due to a perceived risk of treatment‐related complications [8, 9]. Frail patients have reduced physiologic function, increased dependency, increased vulnerability to stressors, and are at increased risk of worse health‐related outcomes [10, 11, 12].

For transplant‐ineligible patients, significant progression‐free survival (PFS) benefit was observed with frontline daratumumab plus lenalidomide and dexamethasone (D‐Rd) versus Rd in the Phase 3 MAIA study (NCT02252172), and D‐Rd set a new benchmark for median overall survival of 7.5 years [7, 13, 14]. An analysis at the 36.4‐month median follow‐up of the subpopulation of patients who were considered frail (based on age, Charlson comorbidity index [CCI], and baseline Eastern Cooperative Oncology Group performance status [ECOG PS] score) showed that D‐Rd efficacy was superior over Rd for PFS (HR, 0.62; *p* = 0.003) [15].

Patients with multiple myeloma (MM) experience significant symptom burden, frequently characterized by fatigue, pain, and impaired day‐to‐day functioning, which impacts patient quality of life [16]. Osteolytic bone lesions are a major underlying cause of pain and reduced mobility, occurring in > 80% of patients with MM [17, 18]. The use of patient‐reported outcomes (PROs) supports a more complete understanding of a treatment's effects from the patient perspective and may be particularly useful for vulnerable populations, including those who are elderly, frail, and/or have bone lesions.

In MAIA, an analysis of patient‐reported health‐related quality of life (HRQoL) over the first 12 months (twelve 28‐day cycles) of treatment showed improvements in pain and global health status (GHS) for patients treated with D‐Rd versus Rd [19]. Here, we present the final analysis of HRQoL results from MAIA, evaluating the long‐term (up to 5 years) effect of D‐Rd versus Rd in transplant‐ineligible patients with NDMM, including in subgroups of elderly patients, frail patients, and those with bone lesions.

## 2 Methods

### 2.1 Study Design

MAIA was a randomized, open‐label, Phase 3 study conducted at 176 sites in 14 countries across the United States, Europe, the Middle East, and Asia Pacific. Study details have been published previously [7]. In brief, transplant‐ineligible patients with NDMM and an ECOG PS of 0–2 were included. Patients were randomized 1:1 to receive D‐Rd or Rd, stratified by International Staging System (ISS) disease stage (I vs. II vs. III), geographic region (North America vs. other), and age (< 75 years vs. ≥ 75 years).

The study was conducted according to Good Clinical Practice guidelines and the Declaration of Helsinki. The study protocol was approved by institutional review boards at all study sites. All patients gave written informed consent.

### 2.2 Treatment

Patients in both treatment groups received dexamethasone 40 mg (20 mg for patients > 75 years) orally or intravenously once a week and lenalidomide 25 mg orally once a day on days 1–21 of each 28‐day cycle, with dose adjustments for renal insufficiency. Daratumumab 16 mg/kg was given intravenously once a week for the first 8 weeks (cycles 1 and 2) of treatment, then every other week for 16 weeks (cycles 3–6), and then every 4 weeks thereafter (cycle 7 and beyond). Treatment continued until disease progression or unacceptable toxicity.

### 2.3 PROs

PROs were assessed using the European Organisation for Research and Treatment of Cancer quality of life questionnaire core 30 (EORTC QLQ‐C30), which comprises a GHS, five functional scales, three symptom scales, and six single items [20]. Patients completed questionnaires electronically at baseline; on Day 1 of cycles 3, 6, 9, and 12; and every 6 cycles thereafter until disease progression. This analysis focuses on the key scales from the EORTC QLQ‐C30 (GHS, physical functioning, pain, and fatigue). These scales were selected based on core symptoms and impacts on patients with myeloma. The recall period was 1 week. Scores were transformed to a scale of 0–100. For GHS and physical functioning scales, higher scores indicate better outcomes whereas for symptom scales of pain and fatigue, lower scores indicate better outcomes [20].

### 2.4 Patient Subgroups

Frail patients were identified using a simplified frailty index based on retrospective evaluation of baseline age, CCI, and ECOG PS score [15, 21]. Patients in the intent‐to‐treat (ITT) population were also stratified according to baseline age of < 70 years, ≥ 70 to < 75 years, and ≥ 75 years. PROs relating to GHS, pain, and physical functioning were also evaluated in the subgroup with baseline bone lesions, as assessed via computed tomography or positron emission tomography–computed tomography.

### 2.5 Endpoints and Statistical Analyses

This final ~5‐year analysis was performed post hoc. Treatment effect was assessed using a mixed‐effects model for repeated measures with the change from baseline score as a dependent variable. Independent variables included baseline score, study visit, assigned treatment, visit by treatment interaction, and randomization stratification factors (for the ITT population) as fixed effects and individual subject as a random effect. Results are shown as least squares (LS) means with 95% confidence intervals (CIs) for each treatment group. Proportions of patients with clinically meaningful improvement were descriptively summarized and compared between arms using Mantel–Haenszel estimate of the common odds. Thresholds for determining the minimally important (clinically meaningful) changes were ≥ 8 points for the EORTC QLQ‐C30 GHS and ≥ 10 points for the EORTC QLQ‐C30 physical functioning, pain, and fatigue scores [22, 23].

For robustness of results, additional analyses of PRO time to worsening used one distribution‐based criterion per PRO scale to define a responder. Worsening was defined as a score change from baseline of at least half a standard deviation, determined from the pooled baseline values of both treatment groups. Time to worsening was defined as the time from randomization to the first observation of worsening summarized using Kaplan–Meier estimate. For patients whose PROs did not worsen, time to worsening was censored at the last PRO assessment. For treatment comparisons, HRs and their 95% CIs were obtained from a Cox proportional hazards model. For ITT analysis, the Cox model includes treatment as the sole explanatory variable stratified with randomization stratification factors (i.e., ISS stage, region, and age). For subgroup analysis, the Cox model includes treatment as the sole explanatory variable.

## 3 Results

### 3.1 Baseline Characteristics and PRO Compliance Rates

Overall, 737 patients were randomly assigned to receive D‐Rd (*n* = 368) or Rd (*n* = 369; Figure S1). Baseline characteristics for the ITT population were balanced across treatment groups [7]. Subgroups included 21% of patients aged < 70 years (D‐Rd, 21%; Rd, 21%), 35% aged ≥ 70 to < 75 years (D‐Rd, 35%; Rd, 36%), 44% aged ≥ 75 years (D‐Rd, 43%; Rd, 44%), and 46% classified as frail (D‐Rd, 47%; Rd, 46%). The majority of patients had bone lesions at baseline (D‐Rd, 70.1%; Rd, 70.7%).

In the ITT population and in subgroups, baseline PRO scores were similar between treatment groups (Table S1). Frail patients tended to have numerically worse baseline scores on all scales compared with the ITT population. Patients who were aged ≥ 75 years tended to have numerically better baseline scores than frail patients. Patients with baseline bone lesions had numerically greater pain compared with the ITT population.

Compliance with PRO assessments was > 80% through cycle 42. Compliance dropped after that timepoint but remained ≥ 59% through cycle 66. Of note, the numbers of patients in each subgroup declined after cycle 36, particularly in the Rd arm.

### 3.2 EORTC QLQ‐C30 GHS

**FIGURE 1:**
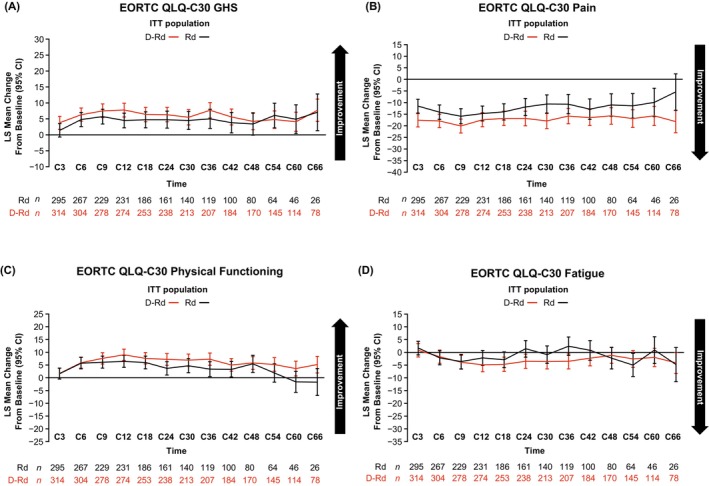
Least squares mean changes from baseline in the ITT population on EORTC QLQ‐C30 GHS (A), pain (B), physical functioning (C), and fatigue (D). C, cycle; D‐Rd, daratumumab plus lenalidomide/dexamethasone; EORTC QLQ‐C30, European Organisation for Research and Treatment of Cancer quality of life questionnaire core 30; GHS, global health status; ITT, intent to treat; LS, least squares; Rd, lenalidomide/dexamethasone.

**FIGURE 2:**
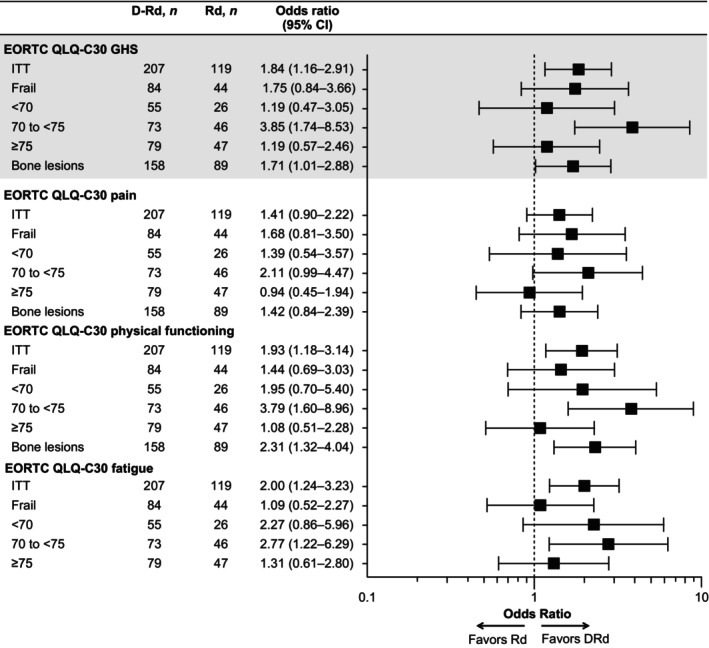
Odds ratios and 95% CIs comparing proportions of patients with clinically meaningful improvement at cycle 36 (year ~3). Clinically meaningful improvement is defined as an increase from baseline ≥ 8 points for EORTC QLQ‐C30 GHS score, ≥ 10 in QLQ‐C30 functional scores, and a decrease ≥ 10 in QLQ‐C30 symptom scores. D‐Rd, daratumumab plus lenalidomide/dexamethasone; EORTC QLQ‐C30, European Organisation for Research and Treatment of Cancer quality of life questionnaire core 30; GHS, global health status; ITT, intent to treat; Rd, lenalidomide/dexamethasone.

In the ITT population, patients in both treatment arms showed sustained improvements from baseline on the EORTC QLQ‐C30 GHS scale at most points throughout the median 64.5‐month follow‐up (Figure 1A). Clinically meaningful improvements were seen in 54.6% and 52.6% of patients in the D‐Rd group at cycles 36 and 60, respectively, versus 39.5% and 50.0% in the Rd group (cycle 36 odds ratio [OR], 1.8 [95% CI, 1.2–2.9]; cycle 60 OR, 1.11 [95% CI, 0.56–2.20]) (Figure 2). Median time to GHS worsening was longer with D‐Rd compared with Rd (26.8 months vs. 21.3 months, respectively; HR, 0.9 [95% CI, 0.7–1.1]).

The findings for the subgroups of frail patients; patients aged < 70 years, ≥ 70 to < 75 years, and ≥ 75 years; and patients with bone lesions were generally similar to the ITT population (Figures S2A–C, S3A, and S4A). For all subgroups, the improvements from baseline in GHS score were similar between treatment arms.

### 3.3 EORTC QLQ‐C30 Pain Subscale

Patients treated with D‐Rd in the ITT population had an ~15‐point improvement in pain scores from baseline throughout the treatment period, exceeding the minimally important change of 10 points (Figure 1B). Improvements in pain were noted in both treatment arms, but a trend for greater improvement in the D‐Rd arm versus the Rd arm was apparent in the ITT population, and across all subgroups (Figures S2D–F, S3B, and S4B). In patients with bone lesions, for whom pain is a particularly important detriment to quality of life, the ~20‐point improvement in pain scores in the D‐Rd arm was still observed (Figure S4B). In this subgroup, proportions of patients with clinically meaningful improvements in pain at cycle 36 were 57.0% for D‐Rd versus 48.3% for Rd (OR, 1.42 [95% CI, 0.84–2.39]), and at cycle 60, were 58.9% versus 42.1% (OR, 1.97 [95% CI, 0.91–4.25]). Median time to worsened pain was 71.5 months with D‐Rd versus 57.5 months for Rd in the ITT population (HR, 0.7 [95% CI, 0.6–0.9]). A longer time to worsening with D‐Rd versus Rd was also seen in the frail (median, not estimable vs. 60.5 months; HR, 0.7 [95% CI, 0.4–1.0]) and aged ≥ 75 years subgroups (median, not estimable vs. 35.0 months; HR, 0.6 [95% CI, 0.4–0.9]).

### 3.4 EORTC QLQ‐C30 Physical Functioning and Fatigue Subscales

On the EORTC QLQ‐C30 physical functioning subscale, mean change from baseline was modest (< 10 points) in both treatment arms of the ITT population (Figure 1C); however, greater proportions of patients in the D‐Rd arm reached the threshold for minimally important change versus the Rd arm at cycle 36 (42.5% vs. 27.7%; OR 1.93 [95% CI, 1.18–3.14]) and cycle 60 (45.6% vs.23.9%; OR, 2.67 [95% CI, 1.23–5.77]) (Figure 2). This trend for patients reaching clinically meaningful change thresholds with D‐Rd was most apparent in patients aged 70 to < 75 years: 47.9% versus 19.6% at cycle 36 (OR, 3.79 [95% CI, 1.60–8.96]) and 55.0% versus 21.1% at cycle 60 (OR, 4.58 [95% CI, 1.29–16.27]) and in patients with bone lesions (cycle 36: 47.5% vs.28.1%; OR, 2.31 [95% CI, 1.32–4.04]; cycle 60: 46.7% vs.23.7%; OR, 2.82 [95% CI, 1.20–6.63]), but sustained score improvements from baseline were seen with D‐Rd across subgroups (Figures S2G–I, S3C, and S4C). A longer time to worsening was noted for the ITT population with D‐Rd than Rd (median, 66.0 vs. 49.3 months; HR, 0.8 [95% CI, 0.6–1.0]), and was also apparent in the frail subgroup (median, 68.1 vs. 39.6 months; HR, 0.66 [95% CI, 0.45–0.95]).

On the EORTC QLQ‐C30 fatigue subscale, mean changes from baseline over time were modest (< 5 points) in both ITT treatment groups (Figure 1D), but the proportion with clinically meaningful improvement was greater with D‐Rd versus Rd at cycle 36 (45.4% vs. 29.4%) and cycle 60 (45.6% vs. 32.6%) (cycle 36 OR, 2.00 [95% CI, 1.24–3.23]; cycle 60 OR, 1.73 [95% CI, 0.85–3.55]); Figure 2). Patients aged 70 to < 75 years had the greatest differences in proportions with clinically meaningful improvement with D‐Rd versus Rd (cycle 36: 46.6% vs. 23.9%; OR, 2.77 [95% CI, 1.22–6.29]; cycle 60: 55.0% vs. 21.1%; OR, 4.58 [95% CI, 1.29–16.27]; Figure 2). Mean changes from baseline were modest across subgroups (Figures S2J–L and S3D).

A negative correlation was found between EORTC QLQ‐C30 physical functioning and fatigue scores at every timepoint throughout the study. Considering patients in both treatment arms, correlation coefficients at baseline (−0.711; nominal *p* < 0.0001), cycle 36 (−0.661; nominal *p* < 0.0001), and cycle 60 (−0.568; nominal *p* < 0.0001) indicated that patients with better physical function report less fatigue.

## 4 Discussion

This final analysis of the MAIA HRQoL data is consistent with the earlier HRQoL findings [19] and extends them through ~5 years of treatment. The addition of daratumumab to Rd in transplant‐ineligible patients with NDMM did not lead to any detriment in HRQoL in the evaluated PROs. Patients treated with D‐Rd had improvements from baseline across PRO scales that were sustained over 5 years, and greater proportions of D‐Rd‐treated patients achieved clinically meaningful improvements versus those treated with Rd, particularly on GHS, physical functioning, and fatigue. Similar findings for sustained improvements were observed for frail and elderly patients with the D‐Rd regimen. Moreover, worsening on each subscale was generally delayed by ≥ 6 months with D‐Rd versus Rd, aligning with the PFS benefit offered by D‐Rd (median PFS, 62 months with D‐Rd vs. 34 months with Rd) [14].

Pain is a characteristic and debilitating symptom of MM that can significantly impact mobility and HRQoL [18]. Pain scores showed a trend for D‐Rd‐treated patients to have greater improvements from baseline versus those treated with Rd. A large (~15‐point) improvement in pain scores was observed with D‐Rd throughout the study; these improvements were greater with D‐Rd versus Rd in terms of mean scores and in the proportion of patients achieving meaningful change, and demonstrate the value of daratumumab in improving this common symptom in patients with MM.

Bone lesions occur in the majority of patients with MM and are associated with significant pain, fractures, and decreased mobility [18]. This was reflected in baseline PRO scores in MAIA, which suggested increased pain and worse physical functioning in patients with baseline bone lesions compared with the ITT population. Importantly, those with bone lesions and treated with D‐Rd showed similar improvements in both pain and physical functioning as patients in the ITT population. These improvements were maintained over the long term with D‐Rd, as shown by the greater proportion of patients with clinically meaningful change, whereas fewer Rd‐treated patients were able to maintain improved scores. Bone pain is a major detriment to quality of life in patients with MM and has been associated with reduced physical, social, emotional, and role functioning, and increased fatigue [24]. Thus, reductions in pain may contribute to improved functioning and overall HRQoL.

MM may limit physical functioning, particularly for elderly and frail patients [25, 26]. We observed improvements with both D‐Rd and Rd in physical functioning in the ITT population. Patients aged ≤ 75 years more frequently had clinically meaningful improvements in physical functioning with D‐Rd than with Rd; a similar trend was noted on the fatigue subscale. Our data on the significant correlation between these two subscales are consistent with previous research that showed that patients with better physical functioning feel less fatigue, and vice versa [20]. Patients aged ≥ 75 years had smaller benefits with D‐Rd on several scales compared with those aged < 75 years. This could be related to effects of lenalidomide in the elderly population, in which the median lenalidomide dose intensity was lower and rates of discontinuation due to adverse events were increased [27].

The current results extend the findings from the 1‐year analysis of HRQoL in the ITT population and in subgroups according to age, ECOG PS, and depth of treatment response [19]. The addition of the frail subgroup in this updated analysis is clinically relevant, as frailty status takes into account patient comorbidities and ECOG PS in addition to age, providing more granularity on patient status, as patient fitness is not dictated by age alone.

The long‐term follow‐up of these PRO results is a strength of this analysis; however, it is limited by its descriptive nature. The low number of patients remaining on study after cycle 42, particularly among the frail and elderly subgroups, is another limitation. In both treatment arms, the patients who remained on treatment long term were likely responding to treatment, which may be associated with improved HRQoL. However, more patients in the D‐Rd arm completed the PRO assessments at the 5‐year timepoint, supporting the efficacy findings of longer PFS with D‐Rd versus Rd over the same follow‐up period (HR, 0.55 [95% CI, 0.45–0.67]; nominal *p* < 0.0001) [14]. Finally, discontinuation of lenalidomide and/or dexamethasone among some patients in the D‐Rd group could have impacted the PRO results [1]; however, assessing this impact was not possible due to the small sample size, variable timepoints of discontinuation, and variability in whether one or both drugs were discontinued.

This updated analysis in transplant‐ineligible patients with NDMM shows durability of the HRQoL improvements that were seen at the 12‐month analysis and indicates that the survival benefit associated with D‐Rd is accompanied by long‐term stability or improvements in HRQoL, including in frail and/or elderly patients.

## Author Contributions

All authors contributed to study design and conceptualization, data interpretation, critically revised the manuscript, approved the manuscript for publication, and agree to be accountable for the integrity of the work. George Wang and Huiling Pei additionally conducted the data analysis.

## Ethics Statement

The study was conducted according to Good Clinical Practice guidelines and the Declaration of Helsinki. The study protocol was approved by institutional review boards at all study sites.

## Consent

All patients gave written informed consent.

## Conflicts of Interest

Aurore Perrot plays a consulting or advisory role in AbbVie, Amgen, BMS, GSK, Johnson & Johnson, Pfizer, Sanofi, and Takeda and received honoraria from AbbVie, Amgen, BMS, GSK, Sanofi, and Takeda. Thierry Facon plays a consulting or advisory role in Amgen, Celgene, Johnson & Johnson, Karyopharm, Oncopeptides, Roche, Sanofi, and Takeda and received speaking fees from Celgene, Johnson & Johnson, and Takeda. Torben Plesner plays a consulting or advisory role in BMS and Celgene. Saad Z. Usmani plays a consulting or advisory role in AbbVie, Amgen, Array Biopharma, BMS, Celgene, GSK, Johnson & Johnson, Merck, Sanofi, Seattle Genetics, SkylineDx, and Takeda Pharmaceuticals, received honoraria from Amgen, BMS, Celgene, Johnson & Johnson, Pharmacyclics, Sanofi, and Takeda Pharmaceuticals, and received speaking fees from Amgen, Johnson & Johnson, and Takeda Pharmaceuticals. Shaji Kumar plays a consulting or advisory role in AbbVie, Celgene, and Kite Pharma and received honoraria from AbbVie, Celgene, and Kite Pharma. Nizar J. Bahlis plays a consulting or advisory role in Amgen and Celgene and received honoraria from Amgen and Celgene. Cyrille Hulin received honoraria from Amgen, BMS, GSK, Johnson & Johnson, Sanofi, and Takeda. Robert Z. Orlowski plays a consulting or advisory role in Amgen, BioTheryX, Celgene, Ionis Pharmaceuticals, Johnson & Johnson, Kite Pharma, Legend Biotech, Molecular Partners, Sanofi‐Aventis, Servier, Spectrum Pharma, and Takeda Pharmaceuticals. Hareth Nahi declares no conflicts of interest. Peter Mollee plays a consulting or advisory role (but no personal fees received) in Amgen, BMS, Johnson & Johnson, and Pfizer and received research funding from Johnson & Johnson and Pfizer. Karthik Ramasamy plays a consulting or advisory role in AbbVie, Adaptive Biotech, Amgen, BMS (Celgene), EUSA Pharma, GSK, Johnson & Johnson, Pfizer, and Takeda, received honoraria from Amgen, Celgene, Johnson & Johnson, and Takeda, and received research funding from BMS (Celgene), GSK, Johnson & Johnson, and Takeda. Murielle Roussel received research funding from Amgen, Celgene, Johnson & Johnson, and Takeda. Arnaud Jaccard plays a consulting or advisory role in Johnson & Johnson, received honoraria from Johnson & Johnson, and received travel expenses from Johnson & Johnson. Michel Delforge plays a consulting or advisory role in Johnson & Johnson and received honoraria from Amgen, BMS, GSK, and Johnson & Johnson. Lionel Karlin plays a consulting or advisory role in Amgen, Celgene, Johnson & Johnson, and Takeda, received honoraria from AbbVie, Amgen, Celgene, Johnson & Johnson, and Takeda, and received travel expenses from Amgen and Johnson & Johnson. Bertrand Arnulf plays a consulting or advisory role in Amgen, Celgene, and Johnson & Johnson, received honoraria from Celgene, Johnson & Johnson, Sanofi, and Takeda, received research funding from Johnson & Johnson, and received travel expenses from Amgen, Celgene, Johnson & Johnson, Sanofi, and Takeda. Ajai Chari plays a consulting or advisory role in Amgen, BMS, Celgene, Johnson & Johnson, Novartis, and Pharmacyclics. George Wang, Niodita Gupta‐Werner, Shuchita Kaila, Huiling Pei, Kathryn Matt, Katharine S. Gries, Robin Carson, and Fredrik Borgsten are employees of Johnson & Johnson, and may own shares/stock options in Johnson & Johnson. Katja Weisel plays a consulting or advisory role in AbbVie, Adaptive Biotech, Amgen, BeiGene, BMS/Celgene, GSK, Johnson & Johnson, Karyopharm, Menarini, Oncopeptides, Pfizer, Roche Pharma, Sanofi, and Takeda, received honoraria from AbbVie, Adaptive Biotech, Amgen, Astra Zeneca, BeiGene, BMS/Celgene, Johnson & Johnson, GSK, Karyopharm, Menarini, Novartis, Oncopeptides, Pfizer, Roche Pharma, Sanofi, Stemline, and Takeda, and received research funding from AbbVie, Amgen, BMS/Celgene, Johnson & Johnson, GSK, Sanofi, and Takeda.

## Supporting information


Table S1.
Figure S1.
Figure S2.
Figure S3.
Figure S4.

## Data Availability

The data sharing policy of Janssen Pharmaceutical Companies of Johnson & Johnson is available at https://www.janssen.com/clinical‐trials/transparency. As noted on this site, requests for access to the study data can be submitted through the Yale Open Data Access (YODA) Project site at http://yoda.yale.edu.
