# Supplementary material for: Sustained Improvement in Health‐Related Quality of Life in Transplant‐Ineligible Newly Diagnosed Multiple Myeloma Treated With Daratumumab, Lenalidomide, and Dexamethasone: MAIA Final Analysis of Patient‐Reported Outcomes
Source: Eur J Haematol. 2025 Feb 14;114(5):883–9. doi: 10.1111/ejh.14392 (PMC11976685; doi:10.1111/ejh.14392)
Supplement: Supplementary file 1 — Table S1. Figure S1. Figure S2. Figure S3. Figure S4. [file EJH-114-883-s001.docx]

**Perrot, et al. Final (5-year) analysis of HRQoL in MAIA**

**SUPPLEMENTAL MATERIALS**

**SUPPLEMENTAL TABLE 1** Baseline EORTC QLQ-C30 scores.^†^

|  | **ITT** | | **Frail** | | **<70 years** | | **70 to <75 years** | | **≥75 years** | | **Bone lesions** | | |
| --- | --- | --- | --- | --- | --- | --- | --- | --- | --- | --- | --- | --- | --- |
| **Mean**  **(SD)** | **D-Rd**  **(*n* = 368)** | **Rd**  **(*n* = 369)** | **D-Rd**  **(*n* = 172)** | **Rd**  **(*n* = 169)** | **D-Rd**  **(*n* = 78)** | **Rd**  **(*n* = 77)** | **D-Rd**  **(*n* = 130)** | **Rd**  **(*n* = 131)** | **D-Rd**  **(*n* = 160)** | **Rd**  **(*n* = 161)** | **D-Rd**  **(*n* = 258)** | **Rd**  **(*n* = 261)** |  |
| GHS | 56.7  (24.8) | 56.2  (24.2) | 50.4  (26.1) | 51.6  (24.3) | 59.7  (23.7) | 54.3  (26.4) | 54.7  (26.0) | 57.7  (25.3) | 56.9 (24.3) | 55.7  (22.2) | 54.5  (25.2) | 55.1  (24.3) |  |
| Pain | 47.4  (36.7) | 44.9  (33.8) | 54.5  (37.8) | 51.8  (34.0) | 48.3  (37.0) | 44.1  (33.2) | 48.3  (35.7) | 44.1  (34.4) | 46.2 (37.5) | 45.9  (33.8) | 52.3  (36.4) | 49.5  (33.3) |  |
| Physical functioning | 63.9  (28.7) | 66.6  (26.1) | 55.2  (29.3) | 57.9  (26.1) | 65.8  (30.2) | 68.5  (28.7) | 63.1  (28.4) | 69.8  (25.1) | 63.7 (28.3) | 63.1  (25.4) | 61.1  (29.9) | 64.9  (26.6) |  |
| Fatigue | 43.5  (29.0) | 42.9  (27.5) | 49.2  (31.4) | 50.2  (27.3) | 42.7  (28.2) | 43.5  (27.0) | 42.9  (28.7) | 38.5  (27.2) | 44.3 (29.8) | 46.0  (27.6) | N/A | N/A |  |

Abbreviations: D-Rd, daratumumab plus lenalidomide/dexamethasone; EORTC QLQ-C30, European Organisation for Research and Treatment of Cancer quality of life questionnaire core 30; GHS, global health status; ITT, intent to treat; N/A, not applicable; Rd, lenalidomide/dexamethasone.
^†^Current analysis focuses on key scores from the EORTC QLQ-C30.

**SUPPLEMENTAL FIGURE 1** CONSORT diagram.


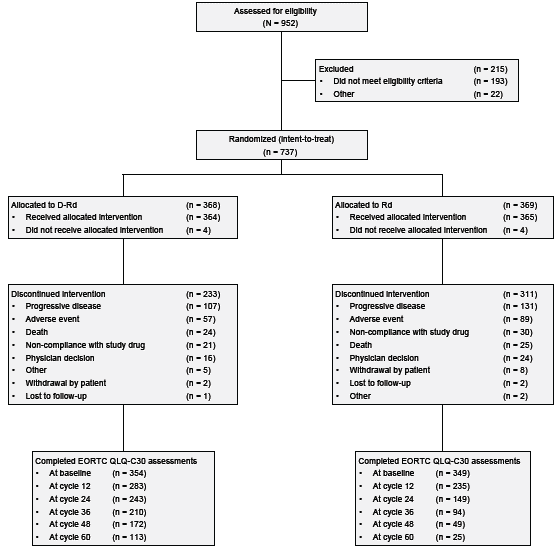


D-Rd, daratumumab plus lenalidomide/dexamethasone; EORTC QLQ-C30, European Organisation for Research and Treatment of Cancer quality of life questionnaire core 30; Rd, lenalidomide/dexamethasone.

Assessed for eligibility

(N = 952)

Excluded (n =215)

- Did not meet eligibility criteria (n = 193)
- Other (n = 22)

Randomized
(n = 737)

Allocated to D-Rd (n = 368)

- Received allocated intervention (n = 364)
- Did not receive allocated intervention (n = 4)

Allocated to Rd (n =369)

- Received allocated intervention (n = 365)
- Did not receive allocated intervention (n = 4)

Discontinued intervention (n = 233)

- Progressive disease (n = 107)
- Adverse event (n = 57)
- Death (n = 24)
- Non-compliance with study drug (n = 21)
- Physician decision (n = 16)
- Other (n = 5)
- Withdrawal by subject (n = 2)
- Adverse event – COVID-19 (n = 1)
- Death – COVID-19 (n = 1)
- Lost to follow-up (n = 1)

Intent-to-treat analysis (n = 368)

- Excluded from safety analysis (n = 4)

Discontinued intervention (n = 311)

- Progressive disease (n = 131)
- Adverse event (n = 89)
- Non-compliance with study drug (n = 30)
- Death (n = 25)
- Physician decision (n = 24)
- Withdrawal by subject (n = 8)
- Lost to follow-up (n = 2)
- Other (n = 2)
- Adverse event – COVID-19 (n = 0)
- Death – COVID-19 (n = 0)

Intent-to-treat analysis (n = 369)

- Excluded from safety analysis (n = 4)

Completed PRO assessments

EORTC QlQ-C30 EQ-5D-5L

- At baseline (n = 354) (n = 349)
- At cycle 12 (n = 283) (n = 273)
- At cycle 24 (n = 243) (n = 233)
- At cycle 36 (n = 210) (n = 201)
- At cycle 48 (n = 172) (n = 164)
- At cycle 60 (n = 113) (n = 111)

Completed PRO assessments

EORTC QlQ-C30 EQ-5D-5L

- At baseline (n = 349) (n = 347)
- At cycle 12 (n = 235) (n = 230)
- At cycle 24 (n = 149) (n = 142)
- At cycle 36 (n = 94) (n = 91)
- At cycle 48 (n = 49) (n = 48)
- At cycle 60 (n = 25) (n = 23)

Assessed for eligibility

(N = 952)

Excluded (n =215)

- Did not meet eligibility criteria (n = 193)
- Other (n = 22)

Randomized
(n = 737)

Allocated to D-Rd (n = 368)

- Received allocated intervention (n = 364)
- Did not receive allocated intervention (n = 4)

Allocated to Rd (n =369)

- Received allocated intervention (n = 365)
- Did not receive allocated intervention (n = 4)

Discontinued intervention (n = 233)

- Progressive disease (n = 107)
- Adverse event (n = 57)
- Death (n = 24)
- Non-compliance with study drug (n = 21)
- Physician decision (n = 16)
- Other (n = 5)
- Withdrawal by subject (n = 2)
- Adverse event – COVID-19 (n = 1)
- Death – COVID-19 (n = 1)
- Lost to follow-up (n = 1)

Intent-to-treat analysis (n = 368)

- Excluded from safety analysis (n = 4)

Discontinued intervention (n = 311)

- Progressive disease (n = 131)
- Adverse event (n = 89)
- Non-compliance with study drug (n = 30)
- Death (n = 25)
- Physician decision (n = 24)
- Withdrawal by subject (n = 8)
- Lost to follow-up (n = 2)
- Other (n = 2)
- Adverse event – COVID-19 (n = 0)
- Death – COVID-19 (n = 0)

Intent-to-treat analysis (n = 369)

- Excluded from safety analysis (n = 4)

Completed PRO assessments

EORTC QlQ-C30 EQ-5D-5L

- At baseline (n = 354) (n = 349)
- At cycle 12 (n = 283) (n = 273)
- At cycle 24 (n = 243) (n = 233)
- At cycle 36 (n = 210) (n = 201)
- At cycle 48 (n = 172) (n = 164)
- At cycle 60 (n = 113) (n = 111)

Completed PRO assessments

EORTC QlQ-C30 EQ-5D-5L

- At baseline (n = 349) (n = 347)
- At cycle 12 (n = 235) (n = 230)
- At cycle 24 (n = 149) (n = 142)
- At cycle 36 (n = 94) (n = 91)
- At cycle 48 (n = 49) (n = 48)
- At cycle 60 (n = 25) (n = 23)

**SUPPLEMENTAL FIGURE 2** Least squares mean changes from baseline for patients <70 years old (**A, D, G, J**), 70 to <75 years old (**B, E, H, K**), and ≥75 years old (**C, F, I, L**).


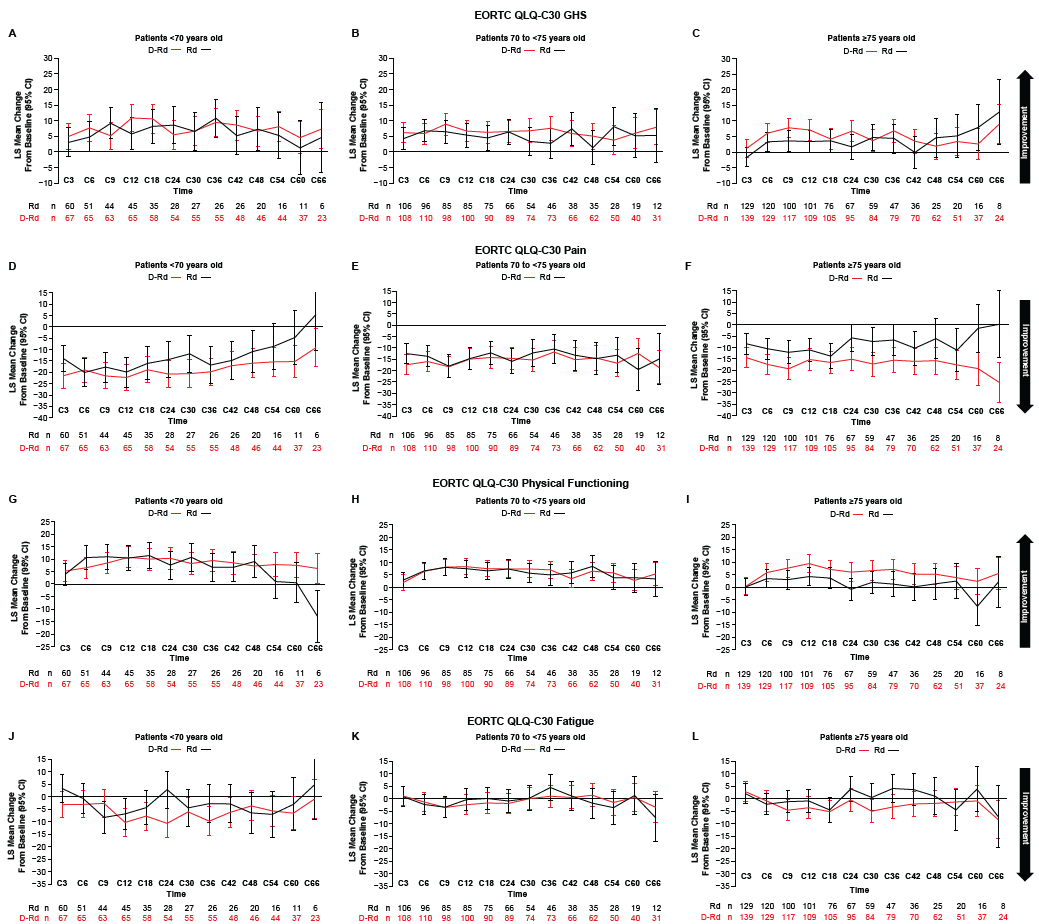


C, cycle; D-Rd, daratumumab plus lenalidomide/dexamethasone; EORTC QLQ-C30, European Organisation for Research and Treatment of Cancer quality of life questionnaire core 30; GHS, global health status; LS, least squares; Rd, lenalidomide/dexamethasone.

**SUPPLEMENTAL FIGURE 3** Least squares mean changes from baseline in EORTC QLQ-C30 GHS (A), pain (B), physical functioning (C), and fatigue (D) in frail patients.


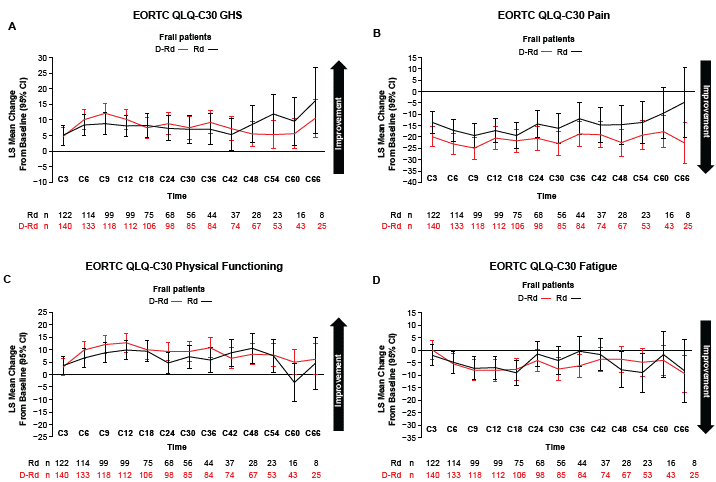


C, cycle; D-Rd, daratumumab plus lenalidomide/dexamethasone; EORTC QLQ-C30, European Organisation for Research and Treatment of Cancer quality of life questionnaire core 30; GHS, global health status; LS, least squares; Rd, lenalidomide/dexamethasone.

**SUPPLEMENTAL FIGURE 4** Least squares mean changes from baseline in EORTC QLQ-C30 GHS (A), pain (B), and physical functioning (C) in patients with bone lesions.


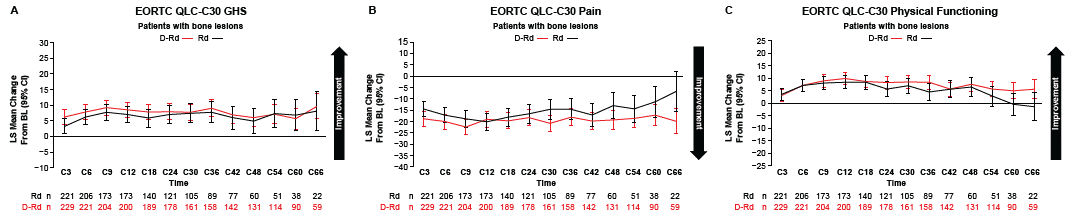


C, cycle; D-Rd, daratumumab plus lenalidomide/dexamethasone; EORTC QLQ-C30, European Organisation for Research and Treatment of Cancer quality of life questionnaire core 30; GHS, global health status; LS, least squares; Rd, lenalidomide/dexamethasone.
